# Does Evenness Even Exist?

**DOI:** 10.1111/ele.70181

**Published:** 2025-07-23

**Authors:** John Alroy

**Affiliations:** ^1^ School of Natural Sciences Macquarie University New South Wales Australia

**Keywords:** compound exponential‐geometric series distribution, evenness, Hill numbers, Hill ratios, Pielou's *J*, Poisson log normal distribution, Shannon's *H*, Simpson's *D*, species richness

## Abstract

The idea that diversity is a combination of species richness and the so‐called “evenness” of count distributions is a bedrock concept in ecology. Researchers often compute stand‐alone evenness indices. They also examine Hill numbers related to Shannon's *H* and Simpson's *D* because these metrics balance richness and “evenness” to various degrees. But evenness is an operationally problematic abstraction, not a thing out in the world. Evenness indices and Hill numbers in empirical data are overly sensitive to the abundance of dominant species, poorly replicable within communities, highly variable among similar communities, and a weak indicator of latitudinal biodiversity trends. They are inconsistently related to the parameters of key models that might underlie count distributions, and they vary highly in simulation even when these model parameters do not vary. Ecologists would benefit by instead determining which real distributions fit which theoretical models and using estimated parameters to understand community structure and assembly.

## Graphical abstract

The idea that diversity is a combination of species richness and abundance distribution evenness is a bedrock concept in ecology. But evenness statistics are highly sensitive to the abundance of individual species, poorly replicable, variable among similar communities, weak indicators of latitudinal trends, and disconnected from ecological processes. A better approach is to quantify variability using the shape parameters of fitted distribution models.

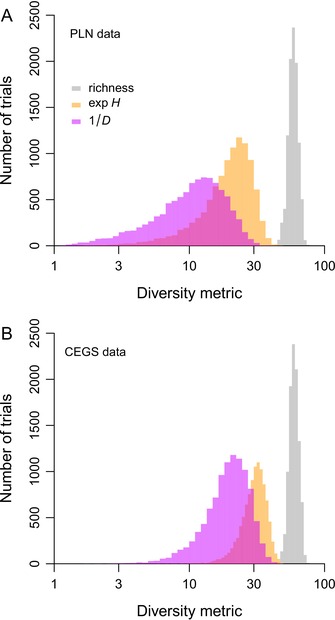

## 1 Introduction

Ecologists say that distributions are “even” when species have similar abundances and “uneven” when one or a few species dominate. In other words, “evenness” is the standard ecological term for variability. This concept is central to the field's history and current practice. It goes back to early work on information theory by Margalef (1957), and the idea was furthered by Pielou (1966) when she defined a simple index called *J* that quantifies it.

*J* is based on the observed number of species *S* and the computed index Shannon's *H*, a metric that summarises how counts of individuals are distributed (Shannon 1948). Specifically, *J* is the ratio *H*/ln *S*, which ranges from 0 to 1 because *H* and *S* are both positive and *H* is equal to ln *S* if and only if all species have identical abundances (Hill 1973). Otherwise, *H* is lower, so the ratio is < 1.

There are numerous other evenness indices, prompting numerous reviews (Hill 1973; Routledge 1983; Smith and Wilson 1996; Heip et al. 1998; Tuomisto 2012; Kvålseth 2015). Not everyone agrees on what an index should look like. For example, Smith and Wilson (1996) were mostly concerned with popularising new indices that were later rarely used; Tuomisto (2012) firmly recommended that evenness must be defined as diversity divided by species richness, but discussed multiple definitions of diversity; and Kvålseth (2015) decided on another rarely used index.

Regardless, the fact that *J* and many other indices range between 0 and 1 was considered a crucial property by certain authors, including Routledge (1983) and Kvålseth (2015). There also does seem to be good agreement with a broad and fundamental suggestion made by early researchers (Lloyd and Ghelardi 1964; Pielou 1966): because “evenness” is a property logically independent of species richness, it is useful to measure. Note the disconnect between the descriptive premise and the normative conclusion.

It has also long been recognised that a spectrum of statistics called Hill numbers (Hill 1973) is potentially informative here because they have blended richness–evenness signals. Hill numbers include *S*; Shannon's *H* after exponentiation; the reciprocal of Simpson's *D* (Simpson 1949; Hurlbert 1971); and the reciprocal of the proportional frequency of the most common species (Berger and Parker 1970). These are respectively called Hill numbers 0, 1, 2, and infinity and notated as 0D, 1D, 2D, and so forth. Fractional and even negative ones are easily computed (Hill 1973; Roswell et al. 2023: Hill number negative infinity is just the count of individuals). Simpson's *D* is particularly important because it is used throughout the social sciences, in genetics, and elsewhere. Meanwhile, Shannon's *H* grounds the field of information theory.

Hill (1973) explained that the above‐mentioned transforms of *H*, *D*, and dominance are “diversity numbers” with units comparable to species counts. He stated that the ratio or log ratio of any two such numbers would be a sensible and dimensionless measure of evenness. For example, e^*H*^ *D* and its log transform *H* + ln *D* compound two numbers with very little sample size bias (unlike *S*: Colwell and Coddington 1994).

Recently, the idea has taken hold that “evenness” signals are so important that it is crucial to explore how the Hill numbers vary in any community (Chao et al. 2014; Moreno et al. 2017; Roswell et al. 2021, 2023). In other words, researchers believe that one should not only count the species *S* but compute diversity numbers based on *H* and *D*. If the motivation for this belief seems unclear, read on.

The Hill number + evenness trend has come to the point where authors like Moreno et al. (2017) and Roswell et al. (2021) suggest not even worrying about the profound sampling bias that plagues *S*. For example, Roswell et al. (2021) pronounced that problem intractable, put aside whether evenness can be measured independent of *S*, and said one should use raw *S* and focus on how changes in *S*, *H* and *D* reflect evenness. Moreno et al. (2017) didn't even mention the idea of extrapolating a true richness value from *S* (Colwell and Coddington 1994). And so things stand.

But justifying this approach depends on making a series of assumptions about the reality and utility of evenness. Here, I argue that it is neither self‐evidently real nor useful on its own, and that a better approach is to directly fit models to the shape of abundance distributions (Connolly et al. 2005; Willis 2019). The distribution fitting strategy disentangles the process of community assembly from the pattern of “evenness”. And as this paper will show, it also permits obtaining largely unbiased richness estimates.

## 2 Materials and Methods

The empirical analyses involve a database of 3095 routine and representative terrestrial species inventories (Alroy 2024). The particular data file was drawn from a public repository called the Ecological Register website (http://ecoregister.org: Alroy 2015, 2019, 2020). The inventories represent trees and groups of animals ranging from mammals to mosquitoes. Every inventory in the data set is matched with integer counts of individuals or sightings of all species present. In addition to the data set's roughly even sampling of a broad array of groups, this fact sets it apart.

The following basic statistics were computed for each inventory: raw richness *S*; Fisher's α (Fisher et al. 1943), an indicator of diversity that considers both sample size and richness and can be translated into the scale parameter *x* of the log series; the basic Hill numbers e^*H*^ (Shannon 1948) and 1/*D* (Simpson 1949), the latter being mathematically more related to so‐called evenness; *J*, nominally a strict measure of evenness; and the most important and reliable logged Hill ratio, *H* + ln *D*. It can also be expressed as ln(e^*H*^ *D*), which might make it clearer that this is the log of the ratio of two species equivalents sensu Hill (1973).

Interpolating the data by coverage‐based rarefaction (Alroy 2010; Chao and Jost 2012) is a widespread alternative approach to the diversity estimation problem. It yields values strongly correlated with *H* and *D*. A variant called iNEXT that extrapolates from rarefaction curves (Chao et al. 2014) has the same problem and is consistently downwards‐biased. Unfortunately, there is no room here to further discuss rarefaction. Readers who are interested are welcome to compare the methods.

Instead, species richness estimates were extrapolated from the data in two ways. First, the counts were fitted to the Poisson log normal abundance distribution (PLN: Bulmer 1974) with the *poilogMLE* function in the *poilog* R package (Grøtan and Engen 2008). The PLN is a keystone distribution with strong properties (Connolly et al. 2005, 2009). Because the PLN's equation specifies the proportion of species with zero counts, it directly implies the total number of species in the community.

Second, the compound exponential‐geometric series distribution (CEGS) was leveraged to do the same thing. This model can be fit using the *cegsML* maximum likelihood‐based function in the *richness* R package (https://github.com/johnalroy/richness).

The PLN and CEGS have analogous properties, but CEGS assumes that underlying abundances are exponentially or Weibull distributed instead of log normally distributed and that the sampling process yielding actual counts is geometric instead of Poisson. It belongs to a large family of what are called compound or mixed species abundance distributions, but the known ones generally assume a Poisson process (Bulmer 1974; Green and Plotkin 2007), and CEGS is apparently new. It is more flexible than the PLN and fits the current data set more closely, but because the focus of this paper is on the evenness concept, its advantages are not dwelt upon.

The distribution's central assumption is that the governing parameter *p* of the geometric series (GS) varies as follows:

(1) $$p=\int 1/\left(E^{\gamma }/\lambda +1\right)\;dE$$

where *p* is the stopping probability of the GS, *E* is a random exponential variate obtained by taking the negative log of a uniform random variate, *γ* is a shape parameter governing variation in counts, and *λ* is an inverse scale parameter governing the magnitude of counts. When *γ* is not 1, *E* ^*γ*^/*λ* is essentially the quantile function of the Weibull distribution, which generalises the exponential. I retain the term “exponential” because it is more familiar, while recognising that CEGS could just as well be called the geometric Weibull distribution to emphasise that it is analogous to the Poisson log normal.

In any event, the CEGS probability mass function is found by plugging *p* into the standard one for the geometric series:

(2) $$P_{X}\left(x\right)={\left(1–p\right)}^{x}\;p$$

where *P*_*X*_(*x*) is the chance that some count *x*, such as 0, 1, or 2, is obtained at random.

The CEGS richness estimator is

(3) $$R=S/\left(1–p\right)$$

where *R* is the overall species pool size. It has modest precision in cases where many values of *γ* and *λ* yield similar likelihoods—and throughout the rest of the text, CEGS parameter estimates are derived using maximum likelihood calculations (*see cegsML*).

Fortunately, precision can be enhanced while paying almost nothing in terms of accuracy by decreasing the influence of outliers. To do this, the counts are slightly compressed by replacing the highest two with the second‐lowest distinct one. For example, a list like 1, 1, 3, 5 and 7 would become 1, 1, 3, 3 and 3 because 1 and 3 are distinct counts. Fitting a model requires the presence of at least three distinct count classes. As a result, at least five species must be present to obtain an estimate here. However, as could be shown with detailed simulations, replacing only the highest value would not improve precision quite as much.

There were five main empirical analyses. (1) Statistics were recomputed after excluding the most common species (= the dominant) from each inventory. This step was taken to show just how strongly the metrics are controlled by a single count, which they should not be because good statistics describe entire distributions and are not beholden to single observations. Furthermore, the Berger–Parker index *d* (Berger and Parker 1970) already captures this precise information. (2) The lists of counts were each randomly and fully resampled with replacement (meaning bootstrapped). A robust statistic should yield the same value after resampling because the number of species is fixed and the average count does not change. (3) Each inventory was matched to the one presenting the most similar sum of reciprocals of counts, and statistics for these pairs were correlated. For example, the count vectors 1, 2, 10 and 1, 2, 1000 are similar because their reciprocals sum to 1 + 1/2 + 1/10 = 1.6 and 1 + 1/2 + 1/1000 = 1.501. Reciprocals and not raw counts were used because the procedure matches inventories with similar numbers of rare species, while leaving the door open for divergent diversity estimates because very common species are largely ignored. (4) The governing shape statistics *σ* of the PLN and *γ* of CEGS were computed for each sample and compared to the two fundamental evenness measures outlined here, *J* and the logged Hill ratio *H* + ln *D*. Real‐world counts have to be generated by some process, so if evenness however defined is unrelated to the shapes of realistic distributions, it presumably says little about processes. (5) Latitudinal gradients in biodiversity were computed for trees and bats. Versions of the same database subsets (Alroy 2024) were used in earlier studies of these groups (Alroy 2019, 2020).

The simulation analyses were based on the PLN and CEGS. In each case, a species pool of 100 was assumed. The first set of 10,000 trials involved random Poisson sampling from communities with log normally distributed sampling rates (= underlying abundances on a continuous scale). This emulated the PLN (Bulmer 1974). Each community had a standard deviation *σ* (meaning shape parameter) of 2, which yields moderately low “evenness”, with an expected median abundance of 1. The second set was drawn from CEGS using the *richness* function *rcegs*. For each sample, the CEGS parameters *γ* (shape) and *λ* (scale) were respectively set to 2 and 1, yielding richness values very similar to those produced by the PLN simulation.

## 3 Results

**FIGURE 1:**
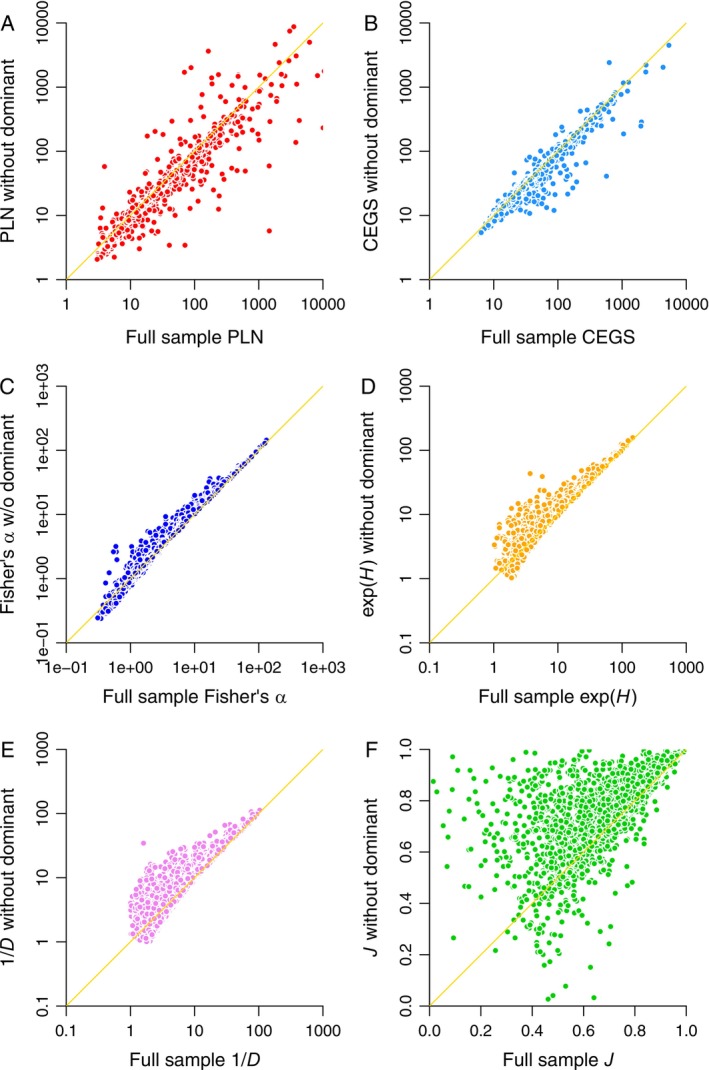
Effects on biodiversity metrics of removing a single dominant species from each species inventory. The data are from the Ecological Register (Alroy 2015, 2024). Estimates based on full inventories are on the *x*‐axis, and those based on the same inventories excluding dominant species, are on the *y*‐axis. Diagonal lines are lines of unity, so estimates yielded by accurate methods should cluster around them. (A) Poisson log normal (PLN) species richness estimates. Spearman's rank‐order correlation coefficient *ρ* = 0.976. (B) Compound exponential‐geometric series (CEGS) richness estimates (*ρ* = 0.982). (C) Fisher's α (*ρ* = 0.990). (D) Shannon's *H*. Values are exponentiated to yield species equivalents (Hill 1973) (*ρ* = 0.943). (E) Simpson's *D*. Values are reciprocal (divided into 1) to yield species equivalents (*ρ* = 0.874). (F) Pielou's *J* (*ρ* = 0.755). For the Hill ratio e^*H*^ *D* (not shown), *ρ* = 0.702.

Removing the dominant species from an assemblage severely distorts Fisher's α, Hill numbers based on *H* and *D*, and evenness measures (Figure 1C–F), while adding noise to PLN richness estimates (Figure 1A) and having no systematic effect on CEGS estimates, despite producing a handful of outliers (Figure 1B: the sample size is 2580).

**FIGURE 2:**
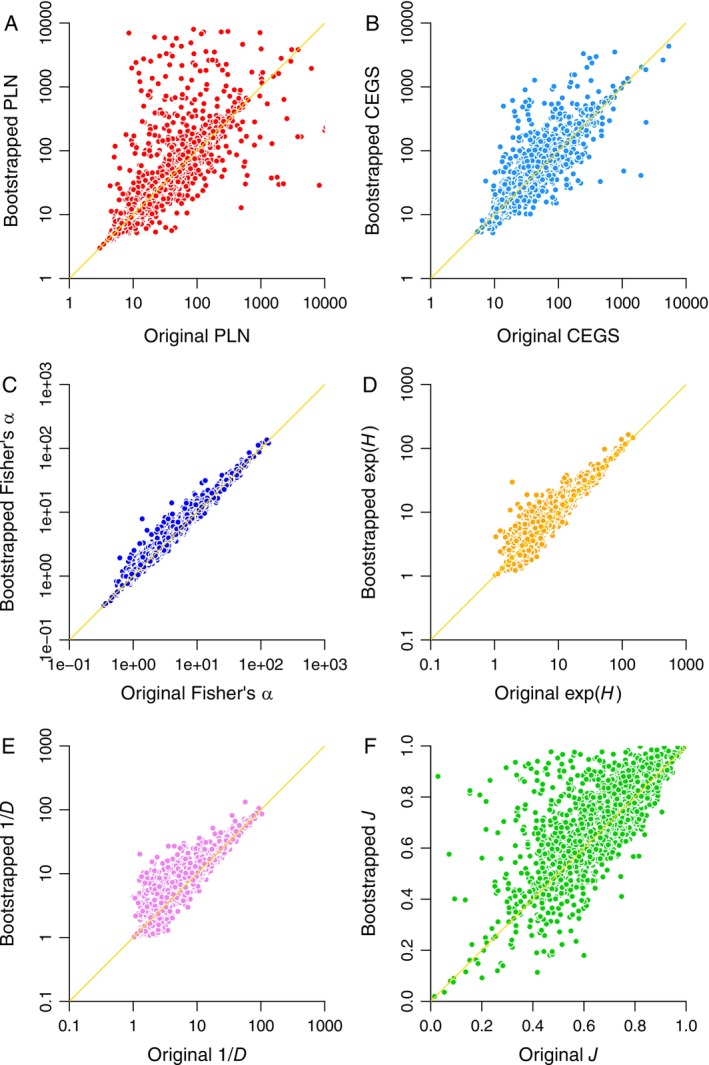
Effects on biodiversity metrics of randomly resampling all of the species within each inventory with replacement (= bootstrapping). Diagonal lines are lines of unity. (A) Poisson log normal (PLN) species richness estimates. Spearman's rank‐order correlation coefficient *ρ* = 0.906; 42.9% of bootstrapped estimates are over the line of unity. (B) Compound exponential‐geometric series (CEGS) richness estimates (*ρ* = 0.916, 45.7% over). (C) Fisher's *α* (*ρ* = 0.985, 51.9%). (D) Shannon's *H* (values are exponentiated: *ρ* = 0.947, 63.1%). (E) Simpson's *D* (values are reciprocal: *ρ* = 0.889, 63.9%). (F) Pielou's *J* (*ρ* = 0.793, 63.1%). For the Hill ratio e^*H*^ *D* (not shown), *ρ* = 0.742 with 32.1% over.

The bootstrap analysis confirms the preceding result by revealing a systematic bias in *H*, *D*, and *J*: resampling the species lists causes all of them to rise (Figure 2D–F). The issue is that all of them are so sensitive to dominant species that randomly omitting or even repeating a high count can cause “diversity” and “evenness” to increase. For example, suppose the counts are 1, 1, 1, and 10. Here, *H* is 2.21. Replacing the high count with a singleton causes *H* to hit 4; duplicating the count and removing a singleton also causes it to increase, now reaching 2.71.

Meanwhile, the two richness indicators are not systematically sensitive to the manipulation, with values consistently centred on the lines of unity (Figure 2A,B). CEGS estimates are more precise (Figure 2B). Fisher's α is even more precise (caption of Figure 2), but numerous values are well above the line of unity (Figure 2C) because it can jump up if a dominant species that is very common is removed.

It is also noteworthy that the raw and resampled Pielou's *J* values are weakly related (Figure 2F). Because the same biological communities are represented each time, this fact raises a serious question as to whether *J* is even a replicable statistic, much less a meaningful one. Previous literature has simply ignored the question of whether *J* and other evenness statistics show consistent patterns when applied to real data (e.g., Tuomisto 2012; Kvålseth 2015).

**FIGURE 3:**
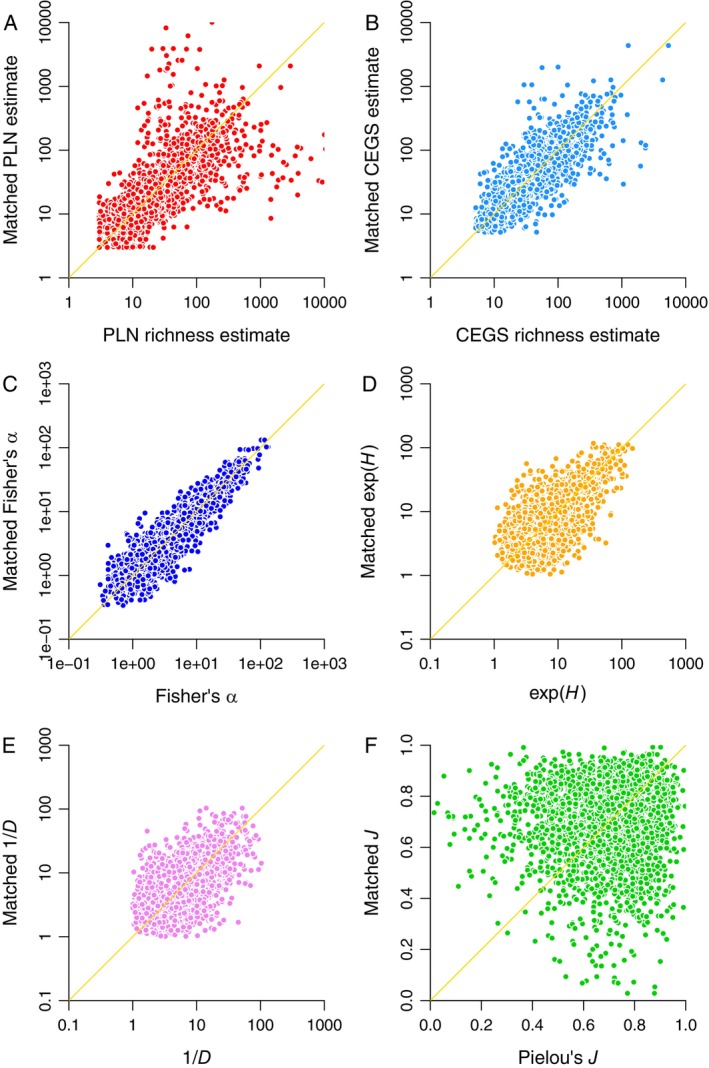
Estimates for matched pairs of species inventories. The members of each pair include a similar number of rare species (see the text). Diagonal lines are lines of unity. (A) Poisson log normal (PLN) species richness estimates. Spearman's rank‐order correlation coefficient *ρ* = 0.843. (B) Compound exponential‐geometric series (CEGS) richness estimates (*ρ* = 0.840). (C) Fisher's *α* (*ρ* = 0.900). (D) Shannon's *H* (values are exponentiated: *ρ* = 0.630). (E) Simpson's *D* (values are reciprocal: *ρ* = 0.499). (F) Pielou's *J* (*ρ* = 0.090). For the Hill ratio e^*H*^ *D* (not shown), *ρ* = 0.276.

Data for samples matched by low counts show much the same thing: high scatter but a definite signal for the PLN (Figure 3A); much less scatter for CEGS and Fisher's α (Figure 3B,C); low scatter but no strong signal for *H* and *D* (Figure 3D,E); and even less of a pattern for *J* (Figure 3F).

**FIGURE 4:**
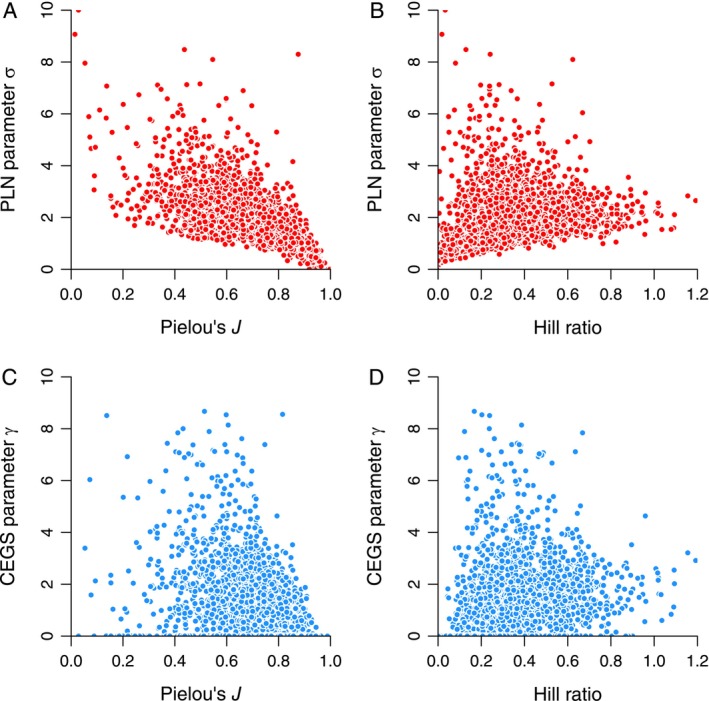
Relationships of Pielou's *J* and the Hill ratio e^*H*^ *D* to the governing shape parameters *σ* of the Poisson log normal (PLN) distribution and *γ* of the compound exponential‐geometric series (CEGS) distribution. Shape parameters determine variability, which “evenness” metrics are supposed to quantify. (A) *J* and *σ*. Spearman's *ρ* = −0.752. (B) The Hill ratio and *σ* (*ρ* = 0.456). (C) *J* and *γ* (*ρ* = −0.404). (D) The Hill ratio and *γ* (*ρ* = 0.376).

Relationships between the respective *σ* and *γ* shape parameters of the PLN and CEGS and either *J* or Hill ratios are subtle at best (Figure 4). This is particularly important because *σ* (top row) equals the underlying standard deviation of the abundances (Bulmer 1974). If anything could ever be called “evenness”, *σ* would qualify. The same argument could be made for *γ* (bottom row), which controls variation in counts when CEGS applies. Both evenness measures do correlate with *σ* and *γ* in a general way, but the relationships are noisy and non‐linear. In particular, the CEGS parameter *γ* is weakly related to either evenness indicator.

Spearman's rank‐order correlation coefficients *ρ* for the comparisons illustrated in Figures 1, 2, 3, 4 are given in the respective captions. Non‐parametric correlations are reported because many of the relationships are non‐linear.

**FIGURE 5:**
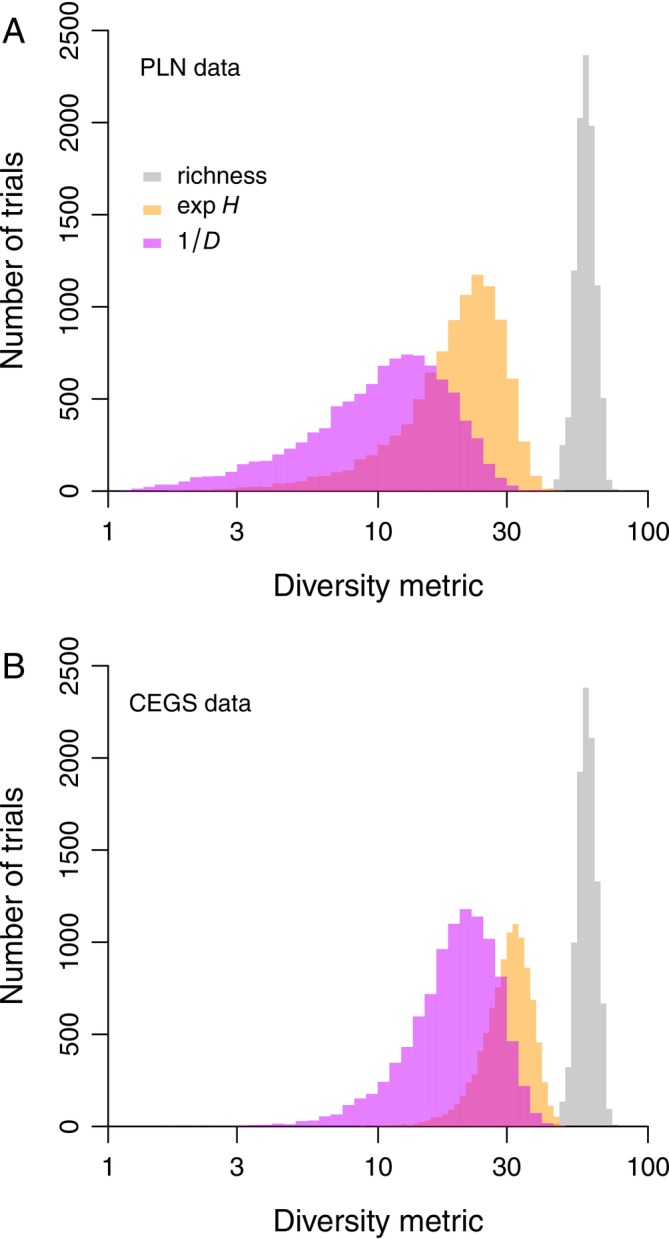
Response of diversity metrics to random variation in counts yielded by two different abundance distribution models. The data are generated by Monte Carlo simulations that assume there are 100 species in each of 10,000 trials. Grey histograms = raw counts of sampled species; orange = Shannon's *H* exponentiated; violet = the reciprocal of Simpson's *D*. (A) The underlying model is the Poisson‐sampled log normal (PLN) with a median draw (*e* ^*μ*^) of 1 individual and a standard deviation (*σ*) of 2 on a log scale. (B) The model is the compound exponential‐geometric series (CEGS) with a scale parameter (*λ*) of 1 and a shape parameter (*γ*) of 2.

The geometric mean sampled richness (*S*) values across 10,000 trials that respectively assumed the PLN and CEGS distributions are 58.6 and 59.4. These figures are realistically close to the underlying true value of 100 that was held constant in every trial (Figure 5). Furthermore, the standard deviations (SDs) of *S* are quite moderate: respectively 0.085 and 0.082 on a natural log scale. By contrast, the species equivalents of *H* and *D* show great spread. For the PLN (Figure 5A), the log‐scale SDs are 0.468 and 0.611, approaching an order of magnitude greater than the value for *S*. For CEG‐distributed community data, the SDs of *H* and *D* are 0.220 and 0.387, still far worse than the SD. of 0.082 for *S*.

**FIGURE 6:**
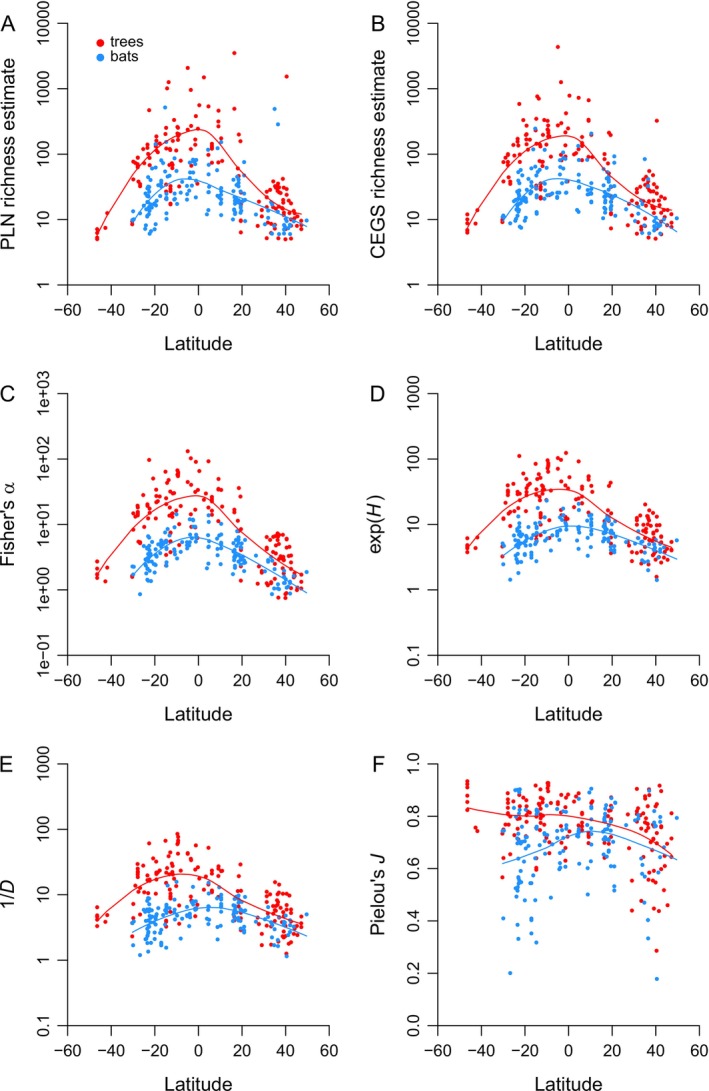
Latitudinal biodiversity gradients in trees (red points) and bats (blue points) from the New World. Lines are non‐parametric and fit using the R function *loess* in the pre‐installed *stats* package. (A) Poisson log normal (PLN) species richness estimates. Standard deviations of logged values (SDs) = 1.850 and 0.840 for trees and bats, respectively. (B) Compound exponential‐geometric series (CEGS) richness estimates. SD = 1.362 and 0.736. (C) Fisher's *α*. SD = 1.270 and 0.601. (D) Shannon's *H* (values are exponentiated). SD = 1.017 and 0.509. (E) Simpson's *D* (values are reciprocal). SD = 0.946 and 0.523. (F) Pielou's *J*. SD values would not be comparable.

Latitudinal diversity gradients in New World trees and bats are clear‐cut when richness and Fisher's α are computed, with peaks near the equator (as expected) and steep climbs near the Tropics of Cancer and Capricorn (Figure 6A–C). Standard deviations of logged values are realistically high (see caption). Shannon's *H* and Simpson's *D* present almost identical trends that depress variability (Figure 6D,E). Pielou's *J* has such high sampling variance that trends are hard to discern and apparently driven by outliers (Figure 6F).

## 4 Discussion

### 4.1 Doesn't Evenness Have to Be Real Because Ecologists Say It Is?

Regular people know that biodiversity is high when species counts are high. They only learn about ecological evenness when they formally study ecology, often well into a tertiary biology degree. Being able to measure something academic does not make it real. For example, we're no longer really talking about MacArthur's broken stick model (MacArthur 1957), even though it was central to the 1960s literature (e.g., Lloyd and Ghelardi 1964). Therefore, most think the broken stick isn't a “real” pattern in nature. Likewise, measuring fractal dimensions went out of vogue about two decades ago, and for good reasons (Halley et al. 2004). If ecologists stopped talking about “evenness” and Hill numbers, would they continue to be real ecological properties?

### 4.2 But If You Can Measure Something Easily, Doesn't That Make It Real?

Statistics like *J*, *H* and *D* can be hand‐calculated. But simplicity is no guarantee of meaningfulness. To be useful, a statistic has to relate to a process out in the world that generates the data.

For example, the arithmetic mean of a true normal distribution is a good estimate of an underlying parameter that generates the data—the process is random generation of values. But the arithmetic mean of a log normal distribution is not, even though the computation is the same. That's because the pattern and process don't match.

To the contrary, abundance distribution parameters like the Poisson log normal's *σ* can be “real” for a given data set, just like the mean and standard deviation of a simple normal distribution truly are. The parameters describe how communities are assembled. So as long as the right model is specified, shape does indicate processes. *J* never does, so it is not a real property of the world.

Similarly, it is easy to count the singletons in a count distribution, but no one would take that seriously as a “diversity” measure because it's obvious that singletons are more common when sampling is relatively poor (Alroy 2010; Chao and Jost 2012). Because sampling is a property of investigation and not of ecological communities per se, a singleton count is an “unreal” pattern description and not a process parameter.

### 4.3 Isn't That Bad Philosophy Because All Numbers Exist?

Sure, but on their own, they are abstract: they can only exist in someone's mind. So, a number by itself doesn't have concrete existence. However, some statistics like the normal distribution's mean can be concrete properties of concrete objects (populations in this case), and not only because they describe how aggregate properties of individual objects are generated from underlying distributions. They are also real because they can cause other properties to change.

For example, temperature is a concrete property of an aggregation of molecules—the mean kinetic energy. Temperature has concrete existence because it causes volume and pressure to change through processes summarised by the ideal gas law. Likewise, changes in mean population densities of organisms can cause changes in mean energy use. And richness is a concrete property that can predict outcomes such as coexistence and extinction cascades (Levine et al. 2017).

In stark opposition, some statistics like Shannon's *H* aren't even intended to describe either sampling processes or causal relations. That is, knowing *H* can never tell you anything concrete because *H* isn't a generating parameter of a distribution, and it's dubious that you can use it to predict changes in ecosystem properties. So it “exists” as a concept but has no utility and is not a concrete property of real populations.

### 4.4 Everyone Agrees on What Evenness Is, So Could That Make It Real?

No, they don't. That's exactly why people keep publishing long review articles about which evenness index is best (e.g., see Tuomisto 2012 vs. Kvålseth 2015). And a key reason is that many of these indices continue to present richness signals. For example, in the original data depicted here (*x*‐axes in Figures 1, 2, 3), the Spearman's rank‐order correlation *ρ* between *S* and *J* is 0.184 with a *p*‐value very near zero. Therefore, Pielou's formula doesn't fully succeed in creating something independent. The reason is that when *S* is undersampled (e.g., see Figure 5), any ratio involving it will indicate more “evenness”.

### 4.5 Couldn't We Just Identify the Best Index and Switch to It?

Maybe, but which one? There's no easy consensus on this point (Tuomisto 2012; Kvålseth 2015), perhaps explaining why authors such as Roswell et al. (2021) aren't actually interested in *J* or other such indices, but instead focus only on Hill numbers. But the latter are intrinsically related to richness because they obey the replication principle (Hill 1973): if true richness doubles and if a Hill number is measured without bias, it too has to double. Therefore, using Hill numbers doesn't actually allow us to measure so‐called evenness on its own.

### 4.6 Couldn't We Solve the Problem by Using Hill Ratios Instead?

The correlation of *S* with the Hill ratio *H* + ln *D* is much higher, and therefore much worse: 0.591. How is this possible? One explanation is that “evenness” is a random outcome of sampling—not only in the sense of reacting strongly to the abundance of dominant species (Figure 1), but even when parameters are held constant across samples (Figure 5). Suppose *D* is close to a random number. Meanwhile, e^*H*^ acts like an average of *S* and 1/*D* almost by definition because it is the Hill number in between them (Hill 1973). Plotting any number *S* against a logged ratio of (1) the average (say geometric mean) of two log normally distributed numbers *S* and 1/*D* and (2) 1/*D* will yield a positive correlation. Keeping in mind that 1/*D* is always less than *S*, this R simulation makes the point:

x < − exp(rnorm(1000)).

y < − exp(rnorm(1000)).

S < ‐ pmax(x,y).

rD < − pmin(x,y).

eH < − (S * rD)^0.5.

Hill.ratio < −log(eH/rD).

cor.test(S, Hill.ratio, method = ‘s’).

So regardless of whether evenness is real, something is wrong with J, and even more so with Hill ratios. Other indices might also suffer from such biases.

### 4.7 Regardless, Aren't Hill Numbers Themselves at Least Highly Replicable?

Perhaps the preceding theoretical problem wouldn't matter if it was at least the case that, given some distribution's specific parameters and some specific richness level, you always got back the same *H* or *D* value in every community. But under simulation, *H* and *D* respond so erratically to random sampling variation that they are virtually uninformative (Figure 5). Keep in mind that none of this variation means anything at all because underlying richness, sampling intensity, and “evenness” (distribution shape) are fixed across simulation trials. What we actually want here is a reliable metric, not something that jumps around. *S* isn't so great in this sense, but *H* and *D* are far worse—not because they are imprecise or because the sample sizes are small, but because they overweight dominant species. Any derivative of *H* or *D*, such as *J* or another Hill ratio, will suffer from the same problem.

### 4.8 Isn't Evenness a Good Proxy for Real‐World Processes?

Abundance distributions can have one, two, or rarely more governing parameters. So one could counter that regardless of replicability, evenness metrics might map closely to some of these parameters. Three well‐known distributions that potentially lie behind many real‐world communities do have two parameters: the Poisson log normal (PLN: Bulmer 1974), negative binomial (NB: Connolly and Thibaut 2012) and Weibull without compounding (Ulrich et al. 2018). There are other previously published examples (Green and Plotkin 2007), and CEGS (Equations 1 and 2) is in the same category. The parameters respectively measure sampling intensity and control the shape of a distribution. If the shape value is high, the distribution is “uneven”, and vice versa. So in principle, there might be a strong relationship between at least one obvious shape parameter and either *J* or the Hill ratios.

There are three key problems with this argument. First, Pielou's *J* is a non‐starter because its sampling variance is too high to yield any real information (Figures 1F, 2F, 3F, 4A,C and 6F). Hill ratios are equally variable or worse (Figure 4B,D and captions of Figures 1, 2, 3).

Second, there are actually only modest correlations between *J* and the Hill ratio *H* + ln *D* on the one hand and the PLN's *σ* parameter and especially the CEGS *γ* parameter on the other (Figure 4). Both determine the variation of counts. Thus, *J* and this Hill ratio have not been shown to stand in for the rules of community assembly: both instead track random variation in dominance (Figures 1, 2 and 5).

Finally, arguing that “evenness” is meaningful on the assumption that the data are specifically generated by some as‐yet unexamined two‐parameter distribution would have to rest on showing that this one distribution fits real data closely. Simply computing the parameters would be more straightforward and would render the concept of evenness obsolete.

There is one limited case where a connection definitely exists. Specifically, Simpson's *D* is a simple function of the *θ* parameter that grounds Hubbell's neutral model of biodiversity (Hubbell 2001; He and Hu 2005). Showing that this model is unrealistic would go far beyond the scope of this paper. Suffice it to say that *θ* is not a variability or shape parameter in the first place, so it makes no difference here how *D* and *θ* might relate.

### 4.9 What About Those Other Distributions?

The current analysis raises a red flag exactly because “evenness” is decoupled from the shape parameters of the PLN and CEGS (Figure 4). And as mentioned, some evenness measure might still be a consistent property of the real world because it might relate to the shape of some other distribution. Which one? A rational ecological model should combine intrinsic variation among species in underlying abundances with a random sampling process (Green and Plotkin 2007). The PLN and CEGS do just that, but other key distributions do not fit the description. For example, the zero‐sum multinomial stems from a population model that ignores sampling (Hubbell 2001); the negative binomial is a pure sampling model; and by itself, the Weibull has no clear model. As discussed, anyone retreating to the argument that the wrong models were tested here would have to show that they are poor. Furthermore, they would have to explain how a favoured alternative is biologically realistic.

### 4.10 What About the Log Series?

Meanwhile, the log series can be predicted either from a sampling process (Fisher et al. 1943) or from population models that ignore sampling (Kendall 1948; Caswell 1976). Its governing parameter α is actually a rescaled version of a second underlying parameter called *x* that simply reflects sampling intensity. So all α really does is combine raw richness and raw sample size according to a standard equation (Chatfield 1969; May 1975). There is nothing in the computation that is really about biology.

More importantly, just by definition, you can't sort out both richness and “evenness” by examining a single parameter like α. The reason is that its equation only considers variation in total richness and total sample size. The log series holds the underlying shape of all distributions constant (Fisher et al. 1943; May 1975), so in a very basic sense it assumes that evenness does not exist.

### 4.11 Isn't There Still Always More to Quantify Than Just Richness?

Just because *J* and Hill ratios are inconsistent measures of shape parameters, that doesn't mean richness is the sole point of interest. Specifically, when a community is actually assembled following a particular distribution having a particular shape parameter, then something other than richness must be measured by definition. But if that's the case, why not just identify this distribution and estimate its shape parameter? Even though the two‐parameter distributions often make similar predictions, there are still reasonable methods for deciding which distribution best fits which data set (e.g., Baldridge et al. 2016).

But is that really all there is to say about count distributions? Indeed yes, because if a distribution like the PLN or CEGS is correctly identified, then nothing else is going on other than scale, shape, richness, and random sampling. Likewise, given a normal univariate distribution, there is nothing to talk about other than the mean, standard deviation, and number of random observations. Meanwhile, if a one‐parameter distribution like the log series is actually the best descriptor of a community, then its parameter is literally the only biological information to be found—and as mentioned, the log series has a fixed shape.

### 4.12 Doesn't Evenness Have a Strong Ecological Signal?

One simple measure of an ecological signal is a correlation with environmental factors. *H* and *D* do reflect simple, strong latitudinal trends (Figure 6D,E), but much more weakly than richness or generalised diversity estimated in any of three ways (Figure 6A–C). There is no indication here that either *H* or *D* tells a different story: they just tell the same story more poorly. Pielou's *J*, meanwhile, again shows so much scatter that it is almost uninformative (Figure 6F).

Hill numbers might still prove to be useful if, say, they do happen to indicate such properties as ecosystem function better than richness does (Roswell et al. 2023). The problem is that to get any such signal, Roswell et al. (2023) had to resort to using negative Hill numbers. As suggested, these numbers essentially average sample size and raw richness, having already stamped out the “evenness” signal of *H* and *D*. Thus, if negative Hill numbers are really all that useful, then “evenness” is all the less useful.

### 4.13 Doesn't Evenness Have a Strong Evolutionary Signal?

Latitudinal diversity gradients are examples of patterns that have deep origins in evolutionary time, maintaining stability for millions of years (Alroy 2010; Mannion et al. 2014). But there is no direct evidence of equally strong latitudinal gradients in any evenness metric—see above. And I have not seen an analysis showing that “evenness” varies with biogeography. Richness does, at least within particular taxa: consider the way the very high richness of different groups in different places has led to the definition of biodiversity hotspots (Myers et al. 2000). It falls on those who take evenness seriously to show that it varies geographically in a clear, consistent manner that says something new and different about evolutionary history, and specifically about truly important patterns like latitudinal gradients.

More importantly, richness is produced by a single process: addition via speciation and immigration and subtraction via extinction and extirpation. When any “evenness” measure is defined independently of a process‐based abundance distribution model, it has no such connection. Instead, it unravels into pure pattern description – a statistic that signifies nothing.

### 4.14 Doesn't Evenness Still Tell Us About Conservation Value?

Perhaps you could ignore all of these issues and assert that because everyone knows Hill numbers are important, they should be used to prioritise conservation (Moreno et al. 2017). Perhaps people should set aside ecosystems with high evenness. Wait, perhaps low evenness. Which one? There are conflicting arguments. In a low evenness community, the rare species are by definition very rare. Thus, they are more likely to disappear without conservation action. But in an “even” community, “biodiversity” is high according to the conventional diversity = richness + evenness definition that goes back at least to Lloyd and Ghelardi (1964). To avoid being hypocritical, ecologists would have to argue that apparently even communities are more valuable. There is no clear resolution to the problem. And in reality, the public wants first and foremost to preserve a wide range of species in their natural habitats, which means preserving richness, not variability.

### 4.15 Don't the Higher Hill Numbers at Least Tell You Different Things About Evenness?

Current literature emphasises presenting multiple Hill numbers on the assumption that the “evenness” signal is weakest for *S*, stronger for e^*H*^, and still stronger for 1/*D* (e.g., Roswell et al. 2021, 2023). But there is no indication that e^*H*^ and 1/*D* yield substantively different patterns. Both are unstable (Figures 1D,E and 2D,E), weakly confirmed by matched data (Figure 3D,E), unable to predict distribution shapes when combined (Figure 4B,D), highly sensitive to randomness in sampling (Figure 5), and suggestive of flattened large‐scale biodiversity trends (Figure 6D,E). Thus, something is wrong not only with raw richness, which everyone has known for decades, but with the idea that high Hill numbers tell different stories from each other because they are progressively less coupled to richness. Backing out an estimate of actual species richness by fitting a distribution or using a robust and well‐justified index, as opposed to just counting up known species, is therefore a better idea.

### 4.16 What Is the Take‐Home Message About Evenness Itself?

The conventional definition of “evenness” comes up short according to every criterion explored here. It is counterintuitive, hard to measure, full of random variation, hard to apply for decision‐making, inconsistently correlated with distribution parameters, and as a result nearly uninformative about the real world. None of these problems are nearly as severe for richness as long as it is correctly estimated. “Evenness” is also a transient attribute with no deep evolutionary history: by contrast, species richness is a long‐term property of biotas that, by definition, is yielded by consistent processes. Granted, ecology's focus on evenness indices and Hill numbers may never subside. But one can hope that many ecologists will consider other strategies for assessing communities.

## Author Contributions

J.A. designed the study, collected the data, created the analytical protocols, analysed the data and wrote the manuscript.

## Peer Review

The peer review history for this article is available at https://www.webofscience.com/api/gateway/wos/peer‐review/10.1111/ele.70181.

## Supporting information


Data S1.

## Data Availability

The data that support the findings of this study are openly available in the Supporting Information and in the Zenodo repository (https://zenodo.org/records/15762247). The underlying Ecological Register data set is available in the Dryad repository (https://doi.org/10.5061/dryad.brv15dvdc). The R code used to carry out the analyses and prepare the text figures is in the Supporting Information and the Zenodo repository (https://zenodo.org/records/15762247). The *richness* package including key functions is on GitHub (https://github.com/johnalroy/richness).
